# Impact of cancer history on clinical outcome in patients undergoing transcatheter edge-to-edge mitral repair

**DOI:** 10.1007/s00392-020-01770-2

**Published:** 2020-11-09

**Authors:** Noriaki Tabata, Marcel Weber, Atsushi Sugiura, Can Öztürk, Kenichi Tsujita, Georg Nickenig, Jan-Malte Sinning

**Affiliations:** 1 Department of Medicine II, Heart Center Bonn, University Hospital Bonn, Venusberg-Campus 1, 53127 Bonn, Germany; 2 Department of Cardiovascular Medicine, Graduate School of Medical Sciences, Kumamoto University, Kumamoto, Japan

**Keywords:** Cancer, Mitral regurgitation, Inflammation, Biomarkers

## Abstract

**Background:**

Little is known about the prevalence of a history of cancer and its impact on clinical outcome in mitral regurgitation (MR) patients undergoing transcatheter mitral valve repair (TMVR).

**Objectives:**

The purpose of this study is to investigate the prevalence of cancer, baseline inflammatory parameters, and clinical outcome in MR patients undergoing TMVR.

**Methods:**

Consecutive patients undergoing a MitraClip procedure were enrolled, and the patients were stratified into two groups: cancer and non-cancer. Baseline complete blood counts (CBC) with differential hemograms were collected prior to the procedure to calculate the platelet-to-lymphocyte ratio (PLR) and neutrophil-to-lymphocyte ratio (NLR). All-cause death within a one-year was examined.

**Results:**

In total, 82 out of 446 patients (18.4%) had a history of cancer. Cancer patients had a significantly higher baseline PLR [181.4 (121.1–263.9) vs. 155.4 (109.4–210.4); *P* = 0.012] and NLR [5.4 (3.5–8.3) vs. 4.0 (2.9–6.1); *P* = 0.002] than non-cancer patients. A Kaplan–Meier analysis revealed that cancer patients had a significantly worse prognosis than non-cancer (estimated 1-year mortality, 20.2 vs. 9.2%; log-rank *P* = 0.009), and multivariable analyses of three models showed that cancer history was an independent factor for 1-year mortality. Patients who died during follow-up had a significantly higher baseline PLR [214.2 (124.2–296.7) vs. 156.3 (110.2–212.1); *P* = 0.007] and NLR [6.4 (4.2–12.5) vs. 4.0 (2.9–6.2); *P* < 0.001] than survivors.

**Conclusions:**

In MitraClip patients, a history of cancer was associated with higher inflammatory parameters and worse prognosis compared to non-cancer patients.

**Graphical Abstract:**

Central Illustration. Clinical outcomes and baseline PLR and NLR values accord-ing to one-year mortality. (Left) Patients who died within the follow-up period had a significantly higher baseline PLR (214.2 [124.2–296.7] vs 156.3 [110.2–212.1]; *P* = 0.007) and NLR (6.4 [4.2–12.5] vs 4.0 [2.9–6.2]; *P* < 0.001) than patients who survived. PLR, platelet-to-lymphocyte ratio; NLR, neutrophil-to-lymphocyte ratio (Right) A Kaplan-Meier analysis revealed that cancer patients had a significantly worse prognosis than non-cancer patients (estimated one-year mortality, 20.2 vs 9.2%; log-rank *P* = 0.009). 
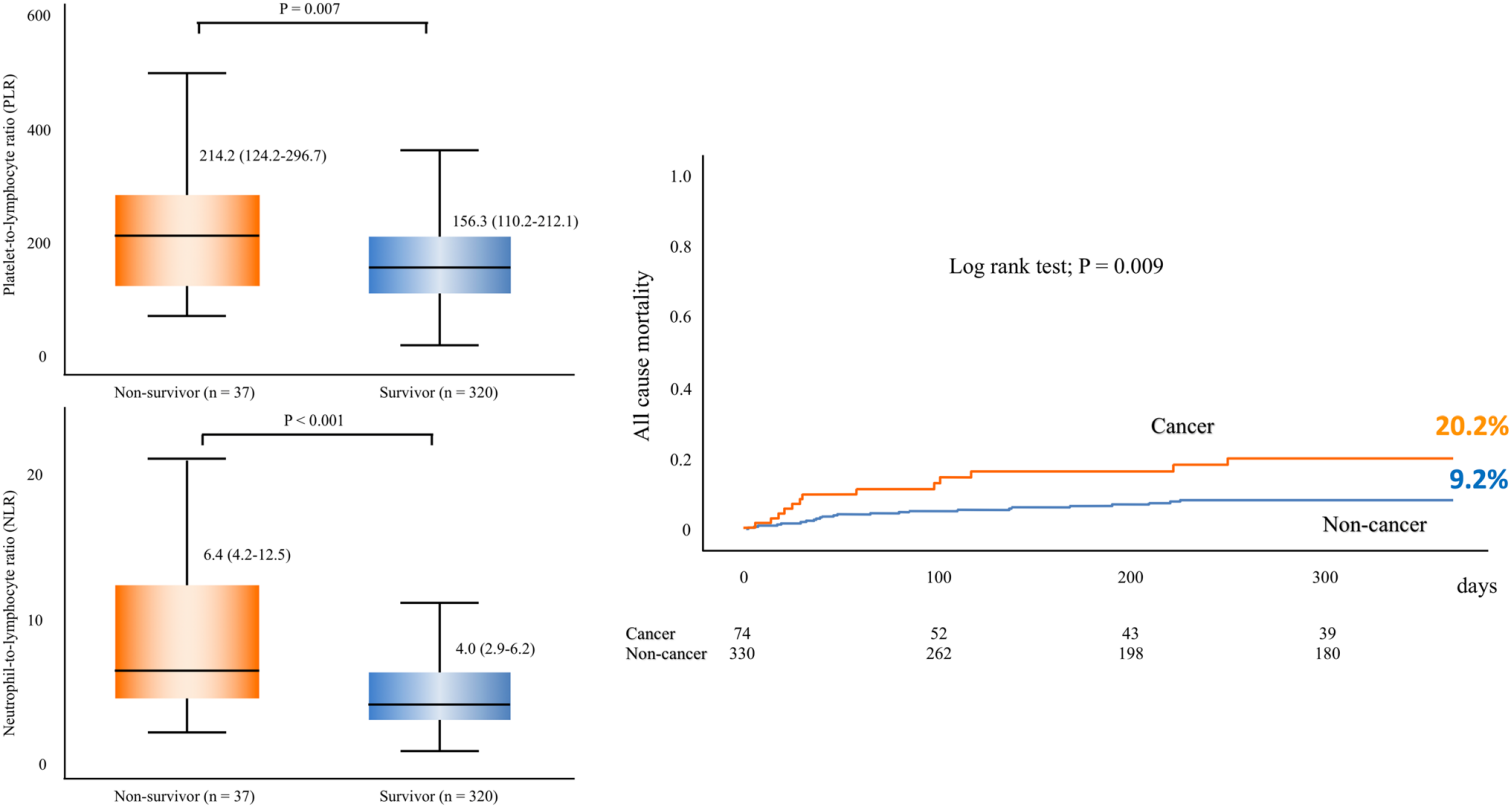

**Electronic supplementary material:**

The online version of this article (10.1007/s00392-020-01770-2) contains supplementary material, which is available to authorized users.

## Introduction

Transcatheter mitral valve repair has emerged as an alternative to surgical mitral valve repair or replacement for symptomatic mitral regurgitation (MR) patients with an increased surgical risk. The most widely used technique is edge-to-edge repair, using the MitraClip system, and its safety and effectiveness have been shown in several studies [1, 2]. Recently, two randomized controlled trials, the MITRA-FR [3] and the COAPT [4] studies, investigated the role of the MitraClip procedures in addition to guideline-directed medical therapy. The COAPT study showed that TMVR using the MitraClip was efficient at reducing heart-failure rehospitalizations as well as mortality, while the MITRA-FR did not demonstrate an effectiveness for the procedure in improving clinical outcomes, since the majority of the patients were already suffering from a too advanced stage of heart failure. Thus, more attention should be paid to which patients would benefit most from this procedure.

The number of cancer survivors continues to increase with advances in early detection and treatment of cancer diseases as well as with the aging of the population [5]. Coexisting cases of cardiac and cancer disease are also increasing and more attention is being given to study this combination, known as the field of “cardio-oncology [6]”. Chronic inflammation is a key pathophysiological cause of both cardiac and cancer diseases [7–9]. Among various inflammatory biomarkers, the platelet-to-lymphocyte ratio (PLR) and the neutrophil-to-lymphocyte ratio (NLR), calculated from complete blood counts with differential hemograms, have been reported to predict clinical outcomes for various types of cancer [10, 11] as well as cardiovascular diseases, including calcific aortic valve diseases [12–14]. Recently, it was reported that patients with a history of cancer undergoing transcatheter aortic valve implantation had a worse prognosis than non-cancer patients [15]. However, little is known about the prevalence of cancer history and its impact on prognosis in MR patients undergoing TMVR.

Therefore, the primary aim of the present study was to compare the PLR and the NLR value of cancer patients with those of non-cancer patients undergoing TMVR using the MitraClip system. The second objective was to evaluate the impact of cancer on the clinical outcome of patients following MitraClip.

## Methods

### Study population and clinical data

This is a single-center, retrospective and observational cohort study. All procedures were conducted in accordance with the Declaration of Helsinki and its amendments and the study was approved by the local ethics committee. Consecutive patients, undergoing TMVR with the MitraClip system between September 2010 and March 2019, were included in this study. Baseline demographic data, previous medical histories, peri-procedural characteristics including echocardiographic parameters, number of implanted clips, and post-procedural residual MR were examined via interview and/or by examining medical records. The European System for Cardiac Operative Risk Evaluation score (EuroSCORE) calculator (https://www.euroscore.org) and the Society of Thoracic Surgery (STS) score calculator (https://riskcalc.sts.org/stswebriskcalc/calculate) were used to calculate the logistic EuroSCORE and STS scores.

### Definition of the cancer group

We divided the patients into two groups according to whether or not they had a history of cancer, called cancer and non-cancer cohorts. A history of cancer was defined as currently having or having a past history of malignant diseases, as previously reported [16]. In the present study, we included both patients that were currently undergoing cancer treatment and/or had any plans to undergo cancer treatments (current cancer) and those that were previously treated for cancer prior to the TMVR procedure (past cancer).

### Complete blood counts with differential

Baseline complete blood counts (CBC) with differential hemograms were collected from a peripheral blood sample obtained prior to the TMVR procedure. The CBC parameters collected were a white blood cell count (WBC), hemoglobin level, hematocrit, and a platelet count; from the WBC count, we further documented three types of leukocytes: neutrophils, lymphocytes, and monocytes. We calculated the ratios between platelets and lymphocytes, platelets and neutrophils, lymphocytes and monocytes, neutrophils and lymphocytes, and neutrophils and monocytes.

### Clinical outcome

The primary endpoint of the present study was all-cause death. After the TMVR procedure, patients were followed up at the outpatient clinic of the University Hospital Bonn or other hospitals, until either the clinical endpoint occurred or the 1-year follow-up was reached. Investigators that were blinded to the study performed the observations, and the information regarding death was ascertained by reviewing the medical records of patients and/or was confirmed by direct contact with the families or physicians of the patients. Patients followed up at other hospitals and/or without follow-up at our hospital were contacted by phone in December 2018 as much as possible.

### Statistical analysis

Continuous variables with skewed distributions are expressed as median values with an interquartile range. Categorical data are presented as numbers and percentages. Differences between the two groups were tested using Fisher’s exact test or a Chi-square test for categorical variables, as appropriate. Differences in continuous variables were analyzed with a Mann–Whitney *U* test. The Kaplan–Meier method was used to estimate the probability of mortality after 1 year, and a log-rank test was performed to compare the distributions of survival times among the groups. Cox proportional hazard analyses were used to calculate the hazard ratio (HR) for clinical outcomes. We performed multivariable analyses in a focused inclusion model. We selected well-known predictors for mortality following the MitraClip procedure [17]. A *P* value <0.05 was considered to denote statistical significance. Statistical analyses were performed using SPSS version 25 (IBM Inc., Armonk, NY, USA).

## Results

### Clinical parameters in study cohorts

**Fig. 1:**
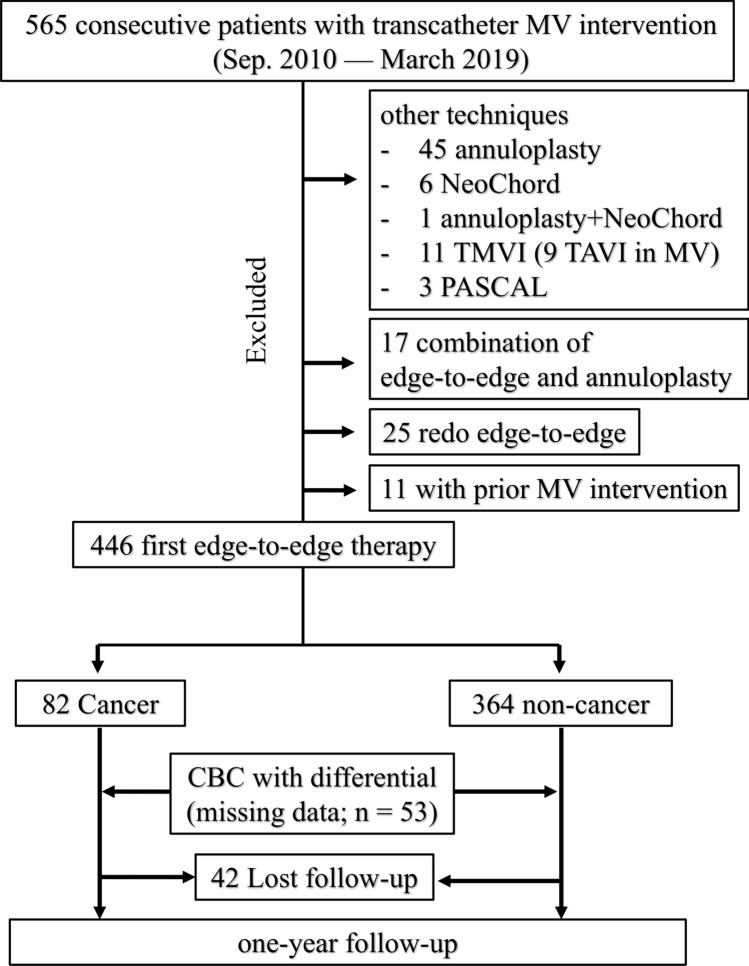
Flowchart of the present study. Out of 565 consecutive patients undergoing transcatheter mitral valve interventions between September 2010 and March 2019, we excluded 66 patients that underwent other techniques than the MitraClip system, 17 patients with edge-to-edge and annuloplasty, 25 patients with redo MitraClip, and 11 patients with a prior mitral valve intervention This resulted in the enrollment of 446 patients that were undergoing their first edge-to-edge therapy using the MitraClip system; among them, 82 patients (18.4%) had a history of cancer. *MV* mitral valve, *TMVI* transcatheter mitral valve implantation, *TAVI* transcatheter aortic valve implantation

Out of 565 consecutive patients undergoing transcatheter mitral valve interventions between September 2010 and March 2019, we excluded 66 patients that were treated with techniques other than the MitraClip system, 17 patients that underwent combined edge-to-edge and annuloplasty procedures, 25 patients with redo MitraClip, and 11 patients with a prior mitral valve intervention. As a result, 446 patients undergoing their first edge-to-edge therapy with the MitraClip system were enrolled in the study (Fig. 1). Among them, 82 patients (18.4%) had a history of cancer (Fig. 1). Cancer types and prior cancer treatments are shown in the Supplemental Table 1; breast (20.7%), colorectal (20.7%), prostate (14%) and leukemia (14%) were the most prevalent cancer types. More than half of the cancer patients (61.0%) had a prior history of surgical cancer treatment (Supplemental Table 1). Thirteen patients had current cancer diseases (stage IV, *n* = 4; stage III, *n* = 2; stage II, *n* = 2; unknown, *n* = 5) and there are four patients undergoing a watchful waiting strategy.

Table 1 compares the clinical characteristics between the cancer and non-cancer groups. At baseline, the clinical parameters were similar between the two groups, except for body mass index (BMI) [24.3 (21.8–27.4) vs. 25.7 (23.1–29.0); *P* = 0.002] and serum levels of N-terminal-pro b-type natriuretic peptide (NT-proBNP) [3751 (2046–8948) vs. 2944 (1325–6622) pg/mL; *P* = 0.018], respectively. Table 2 shows the peri-procedural parameters according to cancer history. Cancer patients had a significantly higher rate of prior aortic valve intervention (17.7 vs. 9.4%; *P* = 0.044). The number of techniques with no clip implantation and post-procedural residual MR were similar between the two groups. Other characteristics were also similar except for medications of the mineralocorticoid receptor antagonist (70.1 vs. 57.3%; *P *= 0.041) and oral hypoglycemic agents (6.5 vs. 15.3%; *P* = 0.045).

**Table 1:** Clinical parameters of the study participants according to cancer history

	Total (*n* = 446)	Cancer (*n* = 82)	Non-cancer (*n* = 364)	*P* value
Male (%)	258 (57.8)	47 (57.3)	211 (58.0)	1.0
Age, years (median range)	79.0 (74.0–83.0)	79.0 (73.0–84.0)	79.0 (74.0–83.0)	0.40
BMI, kg/m^2^ (median range)	25.4 (22.9–28.7)	24.3 (21.8–27.4)	25.7 (23.1–29.0)	0.002
Diabetes (%)	114 (25.6)	14 (17.1)	100 (27.5)	0.051
Hypertension (%)	320 (71.7)	53 (64.6)	267 (73.4)	0.14
Dyslipidemia (%)	237 (53.1)	44 (53.7)	193 (53.0)	1.0
eGFR, mL/min/1.73m^2^ (median range)	47.3 (34.5–57.5)	46.7 (32.1–56.8)	47.4 (34.7–57.6)	0.62
Smoking history (%)				
Current	34 (7.6)	3 (3.7)	31 (8.5)	0.17
Past	72 (16.1)	9 (11.0)	63 (17.3)	0.19
CAD (%)	265 (59.4)	42 (51.2)	223 (61.3)	0.11
Prior PCI (%)	178 (39.9)	29 (35.4)	149 (40.9)	0.38
Prior CABG (%)	111 (24.9)	20 (24.4)	91 (25.0)	1.0
Previous MI (%)	157 (35.2)	26 (31.7)	131 (36.0)	0.52
Previous stroke (%)	47 (10.5)	10 (12.2)	37 (10.2)	0.56
Atrial fibrillation (%)	316 (70.9)	59 (72.0)	257 (70.6)	0.89
PAD (%)	76 (17.0)	11 (13.4)	65 (17.9)	0.42
NYHA (%)				
Class II	55 (12.5)	11 (13.6)	44 (12.3)	0.71
Class III	287 (65.4)	48 (59.3)	239 (66.8)	0.24
Class IV	94 (21.4)	20 (24.7)	74 (20.7)	0.45
Prior device implantation (%)				
Pacemaker	64 (14.3)	13 (15.9)	51 (14.0)	0.73
ICD	62 (13.9)	11 (13.4)	51 (14.0)	1.0
CRT	37 (8.3)	6 (7.3)	31 (8.5)	0.83
COPD (%)	75 (16.8)	13 (15.9)	62 (17.0)	0.87
Logistic EuroSCORE (median range)	15.2 (9.0–23.6)	15.4 (9.6–25.8)	15.2 (8.7–23.2)	0.52
STS score (median range)	3.8 (2.4–6.3)	4.4 (2.8–6.0)	3.7 (2.4–6.4)	0.17
CRP, mg/L (median range)	8.6 (4.4–18.2)	8.7 (4.3–19.2)	8.6 (4.5–18.2)	0.63
NT-proBNP, pg/mL (median range)	3125 (1470–6935)	3751 (2046–8948)	2944 (1325–6622)	0.018

*BMI* body mass index, *eGFR* estimated glomerular filtration rate, *CAD* coronary artery disease, *PCI* percutaneous coronary intervention, *CABG* coronary artery bypass graft, *MI* myocardial infarction, *PAD* peripheral arterial disease, *NYHA* New York Heart Association, *ICD* implantable cardioverter defibrillator, *CRT* cardiac resynchronization therapy, *COPD* chronic obstructive pulmonary disease, *EuroSCORE* the European System for Cardiac Operative Risk Evaluation, *STS score* the Society of Thoracic Surgery Risk Score, *CRP* C-reactive protein, *NT-pro BNP* N-terminal-pro b-type natriuretic peptide

**Table 2:** Peri-procedural parameters according to cancer history

	Total (*n* = 446)	Cancer (*n* = 82)	Non-cancer (*n* = 364)	*P* value
MR etiology (%)				
Primary MR	196 (44.4)	32 (40.0)	164 (45.4)	0.39
Secondary MR	198 (44.9)	42 (52.5)	156 (43.2)	0.14
Mixed MR	47 (10.7)	6 (7.5)	41 (11.4)	0.42
Ejection fraction, % (median range)	48.0 (34.3–60.0)	49.2 (34.6–58.8)	48.0 (33.7–60.1)	0.57
IVS, mm (median range)	1.00 (0.87–1.20)	1.00 (0.83–1.20)	1.00 (0.88–1.20)	0.47
E/eˊ (median range)	17.7 (13.4–22.7)	17.5 (14.5–21.6)	17.7 (13.1–23.5)	0.82
EDV, mm^3^ (median range)	128.3 (91.2–180.8)	125.4 (81.7–174.2)	128.7 (92.0–181.0)	0.49
ESV, mm^3^ (median range)	62.7 (35.5–110.9)	65.6 (37.3–116.5)	60.9 (35.2–110.1)	0.62
LAV, mm^3^ (median range)	92.7 (70.0–124.0)	85.6 (64.6–120.0)	128.7 (92.0–181.0)	0.074
E/e' ratio	17.7 (13.4–22.7)	17.5 (14.5–21.6)	17.7 (13.1–23.5)	0.82
MR quantitative parameters				
PISA, cm (median range)	0.74 (0.63–0.88)	0.71 (0.61–0.87)	0.75 (0.64–0.76)	0.28
VC, cm (median range)	0.62 (0.51–0.74)	0.60 (0.50–0.71)	0.64 (0.51–0.76)	0.10
RV, mm^3^ (median range)	46.7 (35.5–62.0)	45.0 (36.3–60.9)	47.0 (35.0–63.0)	0.75
ERO, cm^2^ (median range)	0.30 (0.20–0.40)	0.32 (0.22–0.41)	0.30 (0.20–0.40)	0.38
Prior AV intervention (%)	46 (11.0)	14 (17.7)	32 (9.4)	0.044
AS ≥ moderate (%)	12 (3.0)	3 (4.5)	9 (2.7)	0.44
AR ≥ moderate (%)	37 (9.3)	7 (10.4)	30 (9.1)	0.82
TR ≥ moderate (%)	278 (62.6)	55 (67.1)	223 (61.6)	0.38
TRPG, mmHg (median range)	43.0 (33.0–52.0)	42.8 (33.0–54.4)	43.0 (32.8–51.9)	0.63
TAPSE, mm (median range)	17.0 (14.0–21.0)	17.0 (13.5–21.0)	17.0 (14.0–21.0)	0.95
Number of clips implanted (%)				
0 clips	26 (5.8)	3 (3.7)	23 (6.3)	0.44
1 clip	188 (42.2)	36 (43.9)	152 (41.8)	0.81
2 clips	202 (45.3)	36 (43.9)	166 (45.6)	0.81
3 clips	30 (6.7)	7 (8.5)	23 (6.3)	0.47
Post-procedural MR ≥ 2 + (%)	126 (29.2)	25 (31.3)	101 (28.8)	0.68
Post-procedural MR ≥ 3 + (%)	35 (8.1)	9 (11.3)	26 (7.4)	0.26
Medications upon discharge (%)				
Aspirin	234 (53.7)	38 (49.4)	196 (54.6)	0.45
P2Y12 inhibitor	270 (61.9)	43 (55.8)	227 (63.2)	0.25
Oral anticoagulant	291 (66.7)	48 (62.3)	243 (67.7)	0.42
Beta blocker	371 (85.1)	63 (81.8)	308 (85.8)	0.38
ARB	118 (27.1)	22 (28.6)	96 (26.7)	0.78
ACE-I	215 (49.3)	35 (45.5)	180 (50.1)	0.53
Diuretics	365 (83.7)	62 (80.5)	303 (84.4)	0.40
MRA	259 (59.5)	54 (70.1)	205 (57.3)	0.041
Statin	279 (64.0)	49 (63.6)	230 (64.1)	1.0
Digitalis	62 (14.2)	12 (15.6)	50 (13.9)	0.72
Oral hypoglycemic agent	60 (13.8)	5 (6.5)	55 (15.3)	0.045
Insulin	23 (5.3)	1 (1.3)	22 (6.1)	0.097
PPI	332 (76.1)	57 (74.0)	275 (76.6)	0.66

*MR* mitral regurgitation, *IVS* interventricular septum, *E/e’* ratio of transmitral Doppler early filling velocity to tissue Doppler early diastolic mitral annular velocity, *EDV* end-diastolic volume, *ESV* end-systolic volume, *LAV* left atrium volume, *PISA* proximal isovelocity surface area, *VC* vena contracta, *RV* regurgitant volume, *ERO* effective regurgitant orifice, *AV* aortic valve, *AS* aortic stenosis, *AR* aortic regurgitation, *TR* tricuspid regurgitation, *TRPG* tricuspid regurgitation peak gradient, *TAPSE* tricuspid annular plane systolic excursion, *ARB* angiotensin receptor blocker, *ACE-I *angiotensin converting enzyme inhibitor, *MRA* mineralocorticoid receptor antagonist, *PPI* proton pump inhibitor

### Platelet-to-lymphocyte ratio between cancer and non-cancer patients

**Fig. 2:**
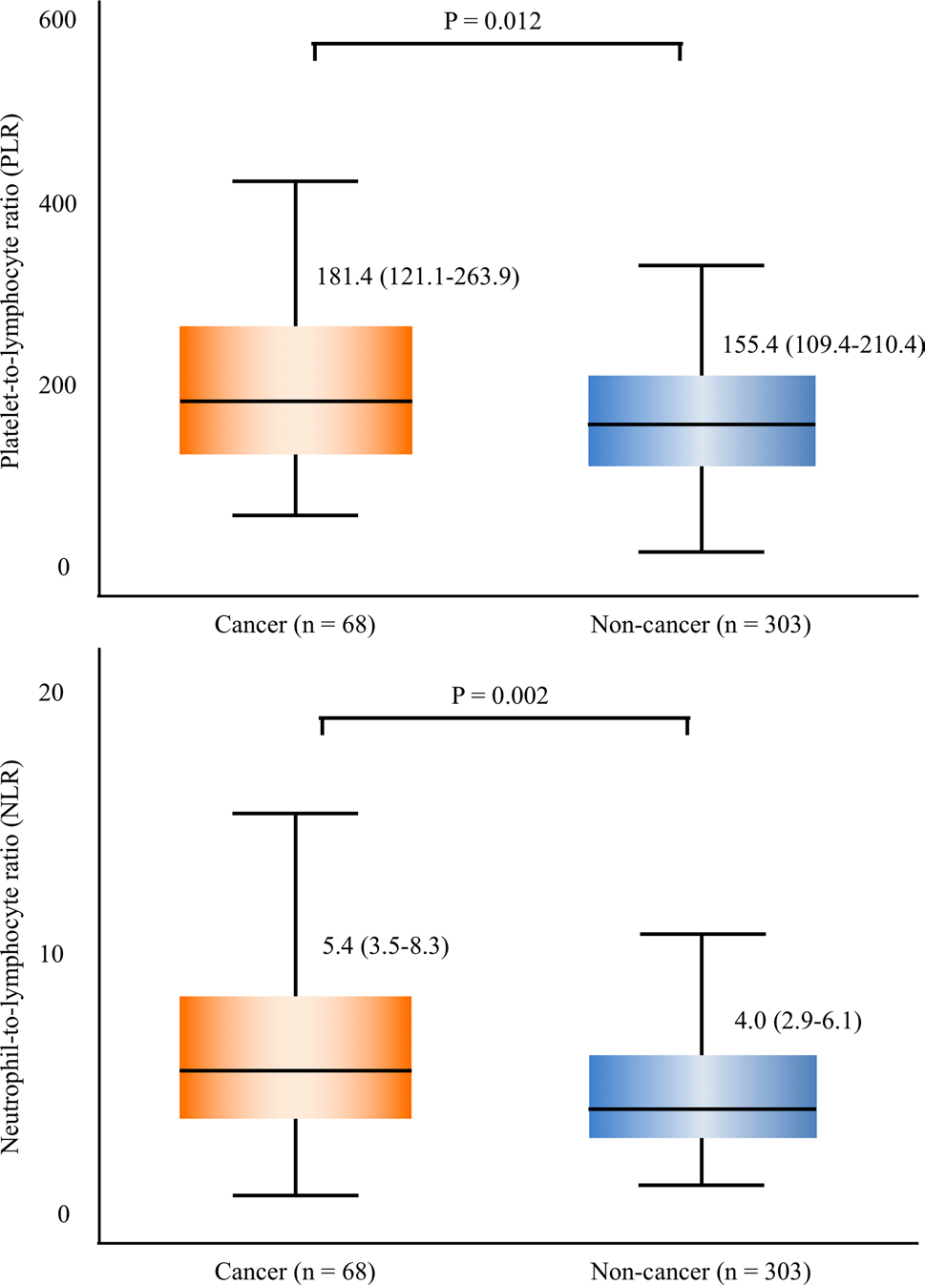
Baseline PLR and NLR between cancer and non-cancer patients. Cancer patients had a significantly higher PLR [181.4 (121.1–263.9) vs. 155.4 (109.4–210.4); *P* = 0.012] and NLR [5.4 (3.5–8.3) vs. 4.0 (2.9–6.1); *P* = 0.002] than non-cancer patients. *PLR* platelet-to-lymphocyte ratio, *NLR* neutrophil-to-lymphocyte ratio

Table 3 shows the baseline values for the CBC with differential hemogram between cancer and non-cancer patients. Cancer patients had a significantly lower level of lymphocytes [1.0 (0.7–1.4) vs. 1.2 (0.9–1.6) × 10^3^; *P* = 0.002] but other parameters, such as neutrophil and monocyte numbers as well as hemoglobin levels, hematocrit, and platelet numbers were similar between the two groups. By calculating the blood cell ratios, we determined that cancer patients had a significantly higher platelet-to-lymphocyte ratio (PLR) [181.4 (121.1–263.9) vs. 155.4 (109.4–210.4); *P* = 0.012] and neutrophil-to-lymphocyte ratio (NLR) [5.4 (3.5–8.3) vs. 4.0 (2.9–6.1); *P* = 0.002] than non-cancer patients. Figure 2 summarizes the key differences in PLR and NLR values between cancer and non-cancer patients.

**Table 3:** Distribution and ratios of differential blood counts and platelet between cancer and non-cancer patients

	Total (*n* = 393)	Cancer (*n* = 68)	Non-cancer (*n* = 303)	*P* value
White blood cells (×10^3^)	7.1 (6.1–8.8)	7.6 (6.3–8.8)	7.0 (6.0–8.8)	0.33
Neutrophils	5.0 (4.1–6.4)	5.3 (4.3–7.4)	4.9 (4.1–6.3)	0.067
Lymphocytes	1.2 (0.8–1.5)	1.0 (0.7–1.4)	1.2 (0.9–1.6)	0.002
Monocytes	0.6 (0.5–0.8)	0.6 (0.4–0.8)	0.6 (0.5–0.8)	0.18
Hemoglobin (g/dL)	11.5 (10.1–13.0)	11.3 (9.9–13.1)	11.6 (10.1–13.0)	0.56
Hematocrit (%)	34.0 (31.0–39.0)	34.0 (30.0–39.0)	34.5 (31.0–38.3)	0.91
Platelets (×10^3^)	181 (149–231)	173 (148–205)	184 (149–232)	0.21
Ratios				
Platelet to lymphocyte	159.0 (111.0–217.1)	181.4 (121.1–263.9)	155.4 (109.4–210.4)	0.012
Platelet to neutrophil	35.2 (27.4–46.6)	33.3 (24.0–40.1)	35.4 (28.0–47.9)	0.029
Lymphocyte to monocyte	1.9 (1.3–2.6)	1.8 (1.2–2.9)	2.0 (1.3–2.6)	0.69
Neutrophil to lymphocyte	4.1 (3.0–6.4)	5.4 (3.5–8.3)	4.0 (2.9–6.1)	0.002
Neutrophil to monocyte	8.1 (6.2–10.1)	8.6 (6.0–13.2)	7.9 (6.2–9.8)	0.13

Values are expressed as median values with an interquartile range (in parentheses)

### Clinical outcome within the one-year follow-up

Out of 446 patients undergoing MitraClip, 42 patients were not available for follow-up after discharge but data for 404 patients were available for determining the clinical outcomes [365 (108–365) days]; of these, a total of 39 patients (9.7%) deceased within the 1-year follow-up. The numbers (Kaplan–Meier estimated probabilities) of cardiac death, non-cardiac death and unknown cause of death between cancer and non-cancer group were 4 (5.9) vs. 8 (2.9) (log-rank *P* = 0.16), 6 (9.2) vs. 14 (5.0) (log-rank *P* = 0.14) and 3 (6.6) vs. 4 (1.5) (log-rank *P* = 0.075), respectively (Supplemental Table 2).

A Kaplan–Meier analysis revealed that cancer patients had a significantly worse prognosis than non-cancer patients (estimated 1-year mortality, 20.2 vs. 9.2%; log-rank *P* = 0.009; Central Illustration, left). The Kaplan–Meier estimated all-cause mortality was 37.5% and 16.6% between patients with current and past cancer diseases, respectively (*P* = 0.19). The Supplemental Figure 1 shows an additional Kaplan–Meier analysis among current cancer, past cancer, and non-cancer patients (among three groups, log-rank *P* = 0.006) and significantly different prognoses among the three groups within a 1-year follow-up period were observed. In the past cancer group, the duration (year) between non-survivors and survivors was 15 (9.5–19.0) and 9.0 (4.0–13.5) (*P* = 0.048), respectively.

Table 4 shows the results of multivariable Cox proportional hazard regression analyses for the prediction of all-cause mortality after the MitraClip procedure. Multivariable analyses were performed using well-known predictive factors for mortality following the MitraClip procedure (model 1: estimated glomerular filtration rate < 30, New York Heart Association IV, NT-proBNP > 5000; model 2: ejection fraction < 30; end-systolic volume > 110; model 3, post-procedural MR ≥ 2, no clip implantation). Using three models, cancer status independently predicted 1-year mortality (model 1: HR, 2.69, *P* = 0.014; model 2: HR 2.60, *P* = 0.008; model 3: HR, 2.23, *P* = 0.029).

**Table 4:** Cox proportional hazards regression analyses for 1-year mortality

Variable	Multivariable regression forced inclusion Model 1			Variable	Multivariable regression forced inclusion Model 2			Variable	Multivariable regression forced inclusion Model 3		
	HR	95% CI	*P*		HR	95% CI	*P*		HR	95% CI	*P*
Cancer	2.69	1.22–5.93	0.014	Cancer	2.60	1.28–5.25	0.008	Cancer	2.23	1.09–4.58	0.029
eGFR < 30	2.23	0.97–5.15	0.060	EF < 30	1.86	0.73–4.73	0.19	Post MR ≥ 3	0.63	0.14–2.86	0.55
NYHA IV	1.48	0.63–3.47	0.37	ESV > 110	0.88	0.37–2.08	0.76	No clip	3.48	0.56–21.63	0.18
NTpro-BNP > 5000	2.53	1.11–5.76	0.028	TR ≥ 2	1.60	0.74–3.46	0.23				

*HR* hazard ratio, *CI* confidence interval, *eGFR* estimated glomerular filtration rate,* NYHA* New York Heart Association, *NT-pro BNP* N-terminal-pro b-type natriuretic peptide, *EF* left ventricular ejection fraction, *ESV* left ventricular end-systolic volume, *TR* tricuspid regurgitation, *Post MR* post-procedural mitral regurgitation

Patients who died within the follow-up period had a significantly higher baseline PLR [214.2 (124.2–296.7) vs. 156.3 (110.2–212.1); *P* = 0.007] and NLR [6.4 (4.2–12.5) vs. 4.0 (2.9–6.2); *P* < 0.001] than patients who survived (Central Illustration, right). The PLR and NLR between non-survivors and survivors are 222.3 (102.6–395.5) vs. 168.1 (117.9–263.9) (*P* = 0.40) and 6.1 (4.9–20.2) vs. 5.4 (3.3–8.3) (*P* = 0.13) in the cancer group (Supplemental Figure 2A) and 190.0 (127.7–270.3) vs. 153.3 (109.0–206.2) (*P* = 0.017) and 6.4 (4.0–10.4) vs. 3.9 (2.8–5.8) (*P* < 0.001) in the non-cancer group (Supplemental Figure 2B), respectively.

## Discussion

In the present study, we investigated the impact of cancer on clinical outcome in patients with symptomatic MR undergoing a MitraClip procedure. The main findings of the present study were as follows: (1) out of 446 MR patients undergoing a first edge-to-edge therapy, 82 patients (18.4%) had a history of cancer; (2) cancer patients had significantly higher baseline PLR and NLR than non-cancer patients; (3) at 1-year follow-up, MR patients with a history of cancer had a significantly worse prognosis than those without cancer, and a cancer history was found to be an independent predictor for 1-year mortality; (4) patients who died within 1 year had significantly higher baseline PLR and NLR than patients who survived.

### Prevalence of cancer diseases in TMVR patients

In our cohort that underwent TMVR for symptomatic MR, 18.4% of patients had a history of cancer. With the aging of the population and improvements in early detection and treatment of cancer diseases, coexisting cases of cardiac and cancer disease is ever increasing [18]. A previous survey in the United States reported that around 20% of all cancer survivors suffer from cardiac diseases and that the co-existence of the two diseases was more prominent in older subjects [18]. Currently, TMVR is generally performed in elderly patients, as shown in our cohort (median age, 79.0 years), which predisposes them to a higher rate of coexisting cancer diseases. The present cohort included "all-comer" TMVR patients and the high frequency of cancer diseases likely reflects the real clinical situation.

Clinical characteristics were mostly similar between the patients with and without a history of cancer, but cancer patients did have a significantly lower BMI and higher NT-pro BNP levels. The lower BMI in cancer patients, despite no significant difference in age or sex, might be indicative of previous cancer treatments such as surgery, chemotherapy, and radiotherapy, as shown in the Supplemental Table1, which could potentially predispose cancer patients to be frailer. Although more prior aortic valve interventions were observed in the cancer group, there were no significant differences in left ventricular ejection fraction, left ventricular end-diastolic volume, left ventricular hypertrophy and diastolic function as well as MR etiologies between the cancer and non-cancer patients. There is also a possibility that cancer status might have caused elevated plasma BNP levels due to cancer-related inflammation according to a previous report [19].

### PLR and NLR in cancer patients

In the present study, cancer patients had significantly higher baseline PLR and NLR levels than non-cancer patients (Table 3). It is well known that a systemic inflammatory response is a critical component of the progression of cancers [8, 20]. Among various inflammatory parameters, the platelet-to-lymphocyte ratio (PLR) and neutrophil-to-lymphocyte ratio (NLR) have been well studied and proposed as markers to predict the clinical outcomes of various cancer types [21–23].

To date, various studies have investigated the role of platelets, neutrophils, and lymphocytes with regard to the progression of cancer. Platelets play an important role in cancer progression by increasing angiogenesis through the action of the cytokine vascular endothelial growth factor [24]. Neutrophils can also promote tumor growth and metastasis through the release of cytokines [25]. In contrast, lymphocytes play an important role in the suppression of cancer by inducing apoptosis in cancer cells [26], and granulocytes have been reported to inhibit the function of cytotoxic lymphocytes [27].

From this point of view, the ratios of platelets-to-lymphocytes and neutrophils-to-lymphocytes can offer a comprehensive evaluation of host immunological reactions. To the best of our knowledge, this is the first report to reveal that patients with a cancer history undergoing TMVR had higher baseline values of PLR and NLR than those without a cancer history. Pre-procedural evaluation of CBC with differential hemogram might be useful to examine the baseline inflammatory status of MR patients undergoing TMVR. It might seem strange that in a condition of a higher chronic inflammatory status measured by PLR and NLR, C-reactive protein, another marker of inflammation, did not show significant differences. C-reactive protein has been widely used to assess inflammatory status and has been found to be strongly associated with the risk and prognosis of cardiac diseases [28]. However, it has also been reported that PLR and NLR may be superior to CRP [29] and that even with the addition of CRP in a multivariable model, NLR predicted clinical outcome in the general population [30]. Therefore, the inflammatory pathways of PLR/NLR and CRP and their impact might be different, although both parameters are useful in inflammatory markers.

### Clinical outcomes in cancer patients undergoing TMVR

TMVR is normally performed in patients who are deemed to be at a high surgical risk due to their age and/or comorbidities. Cancer survivors might be at higher risk for surgical mitral valve repair or replacement because of factors such as mediastinal fibrosis, severe lung disease, prior thoracic surgeries, chest radiation, and/or greater frailty. However, there is little data yet with regard to clinical outcomes in cancer patients undergoing TMVR.

The results of the present study revealed a worse prognosis after TMVR for patients with a history of cancer. Moreover, the Supplemental Figure shows that both current and past cancers had a negative impact on clinical outcomes. As this is the first report to investigate the impact of cancer on clinical outcomes in TMVR patients, there is no clear explanation. However, we could speculate the following: First, patients with a history of cancer have received intensive treatments including surgical, chemo-, and radiation therapy, as was also seen in our cohort (Supplemental Table 1). These previous cancer treatments might predispose TMVR patients to be more frail than non-cancer patients. Interestingly, longer duration between cancer therapy and TMVR was statistically associated with 1-year mortality. There were no significant correlations between the duration and other parameters in the present study. Because the number of past cancer patients is low, further study including large number of subjects would be required to clarify this result. Second, the outcome of death in cancer patients might be due to the cancer disease itself. Although most of TMVR patients were not currently receiving treatment for cancer at the time of TMVR, some patients might have suffered from a recurrence of their cancer. Third, the underlying chronic inflammation might have directly led to a poor prognosis in cancer patients. In the present study, patients who deceased within the 1-year follow-up had significantly higher baseline values for PLR and NLR. It has previously been recognized that chronic inflammation is associated with prognosis of cardiac diseases including coronary artery diseases and chronic heart failure [31, 32]. Moreover, a previous study has shown that the PLR and NLR were higher in patients with heart failure and that the NLR predicted future mortality in heart-failure patients [33]. Thus, the higher degree of inflammation in cancer patients might be associated with a poor prognosis following TMVR.

Both PLR and NLR values are rather uncommon and have been rarely used in clinical practice. However, hemograms with differential are routinely examined and the ratios of platelet to lymphocyte and neutrophil to lymphocyte are easy to calculate. Moreover, these values have been well studied and proposed as markers to predict the clinical outcomes of various cancer types. Thus, these markers would be potential useful surrogate marker in the future clinical practice considering that the number of cancer survivors is expected to increase with advances in early detection and treatment of cancer diseases as well as with the aging of the population. As other confounding factors such as smoldering infections and small inflammatory reactions induced by cardiac catheterization, additional data are warranted to confirm our findings. Results of the present study remain hypothesis generating, and PLR and NLR values should currently not be used for patient selection in clinical practice until they are verified in a larger patient cohort.

### Study limitations

The present study has several limitations. First, this is a single center and observational cohort study, which included a relatively small number of patients undergoing TMVR. Second, we included "all-comer" TMVR patients but excluded patients that were missing a differential white blood cell count or follow-up data, which could lead to some bias. Third, we included a broad spectrum of cancer disease, irrespective of stage and potential cardiotoxic treatment and distinct scenarios such as chemotherapy and advanced diseases that could influence outcome after MitraClip were not investigated. Also, the type of cancer treatment could influence cardiovascular mortality by cardiotoxic side effect [34], but sufficient data of the detailed chemotherapy were not available. Fourth, the roles of PLR and NLR as a predictor of mortality have already been shown for heart failure, thereby limiting the novelty of this finding considering that most patients undergoing the MitraClip procedure suffer from heart failure. Fifth, the study investigated all-cause mortality instead of cardiovascular mortality as a primary endpoint. Therefore, it could be determined whether presence of cancer influences cardiovascular mortality or the increased mortality is simply induced by deaths from cancer itself. Additionally, more severe cardiac condition in the cancer patients indicated by higher NT-proBNP, more aortic valve interventions and higher frequency of mineralocorticoid receptor antagonist use, might have influenced clinical outcome. Furthermore, we did not investigate the impact of the mitral regurgitation in a steady state several months after TMVR. Finally, present study evaluated only the PLR and NLR value and did not evaluate other markers like procalcitonin, cytokines and chemokines. Thus, additional pathophysiological and molecular physiological data still need to be collected.

### Conclusions

In MitraClip patients, cancer patients were associated with higher inflammatory parameters and worse prognosis than non-cancer patients.

## Electronic supplementary material

Below is the link to the electronic supplementary material.

Electronic supplementary material 1 (TIFF 26370 kb)

Electronic supplementary material 2 (TIFF 26370 kb)

Electronic supplementary material 3 (TIFF 26370 kb)

Electronic supplementary material 4 (DOCX 30 kb)

Electronic supplementary material 5 (DOCX 28 kb)
