# Supplementary figures and images for: Impact of cancer history on clinical outcome in patients undergoing transcatheter edge-to-edge mitral repair
Source: Clin Res Cardiol. 2020 Nov 9;110(3):440–50. doi: 10.1007/s00392-020-01770-2 (PMC7907025; doi:10.1007/s00392-020-01770-2)

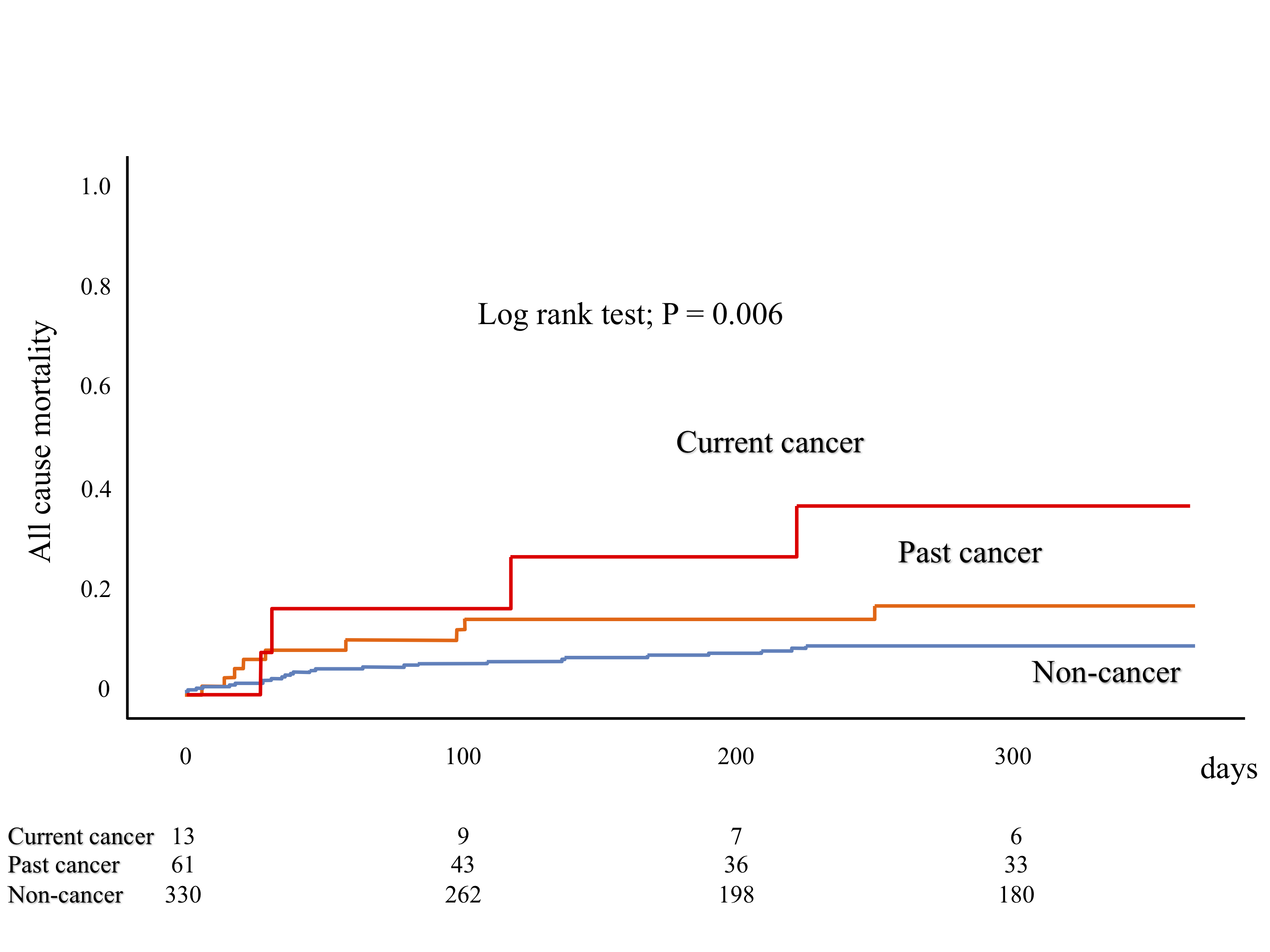

Supplement: Supplementary file 1 — Electronic supplementary material 1 (TIFF 26370 kb) [file 392_2020_1770_MOESM1_ESM.tiff]

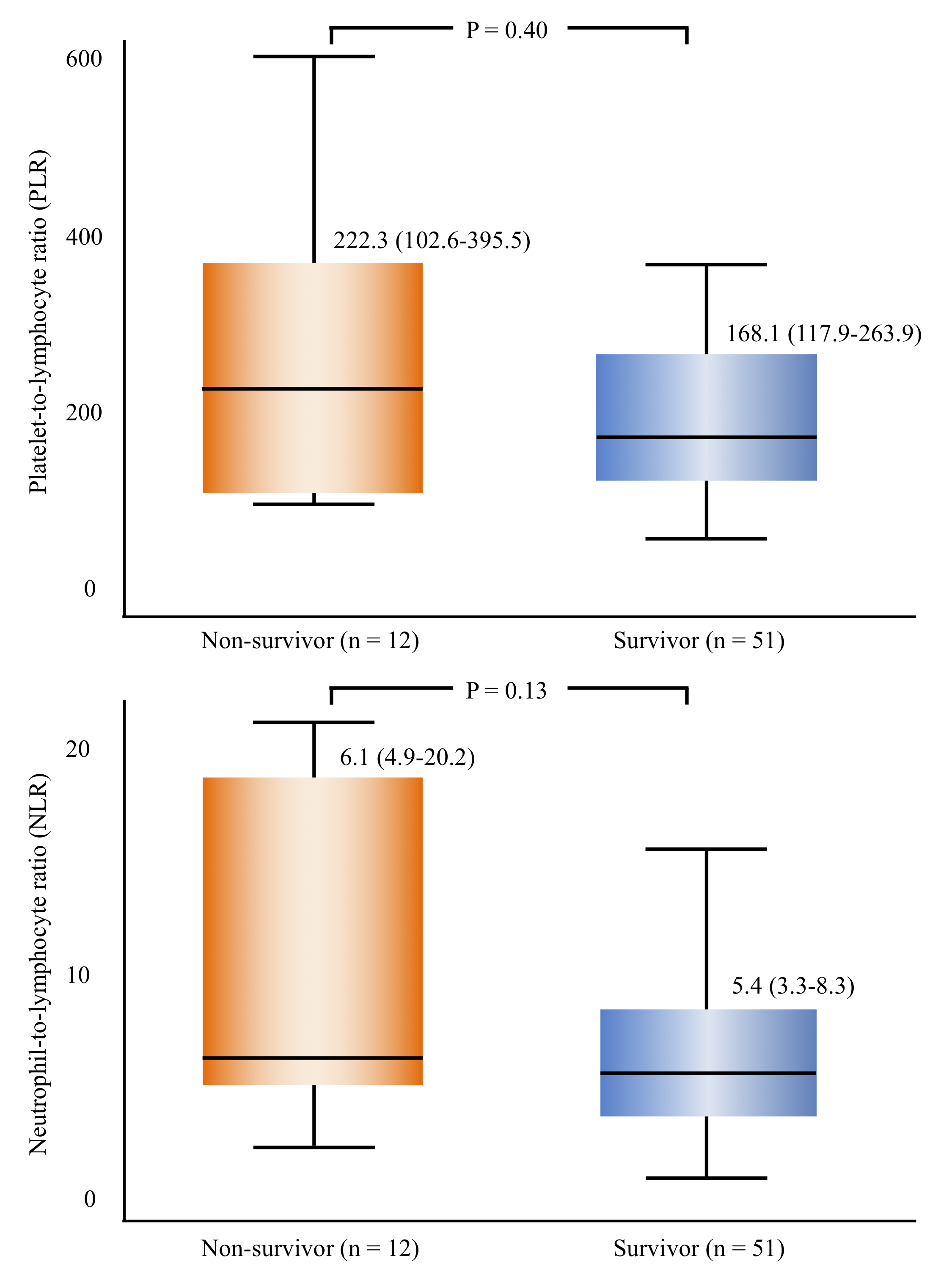

Supplement: Supplementary file 2 — Electronic supplementary material 2 (TIFF 26370 kb) [file 392_2020_1770_MOESM2_ESM.tiff]

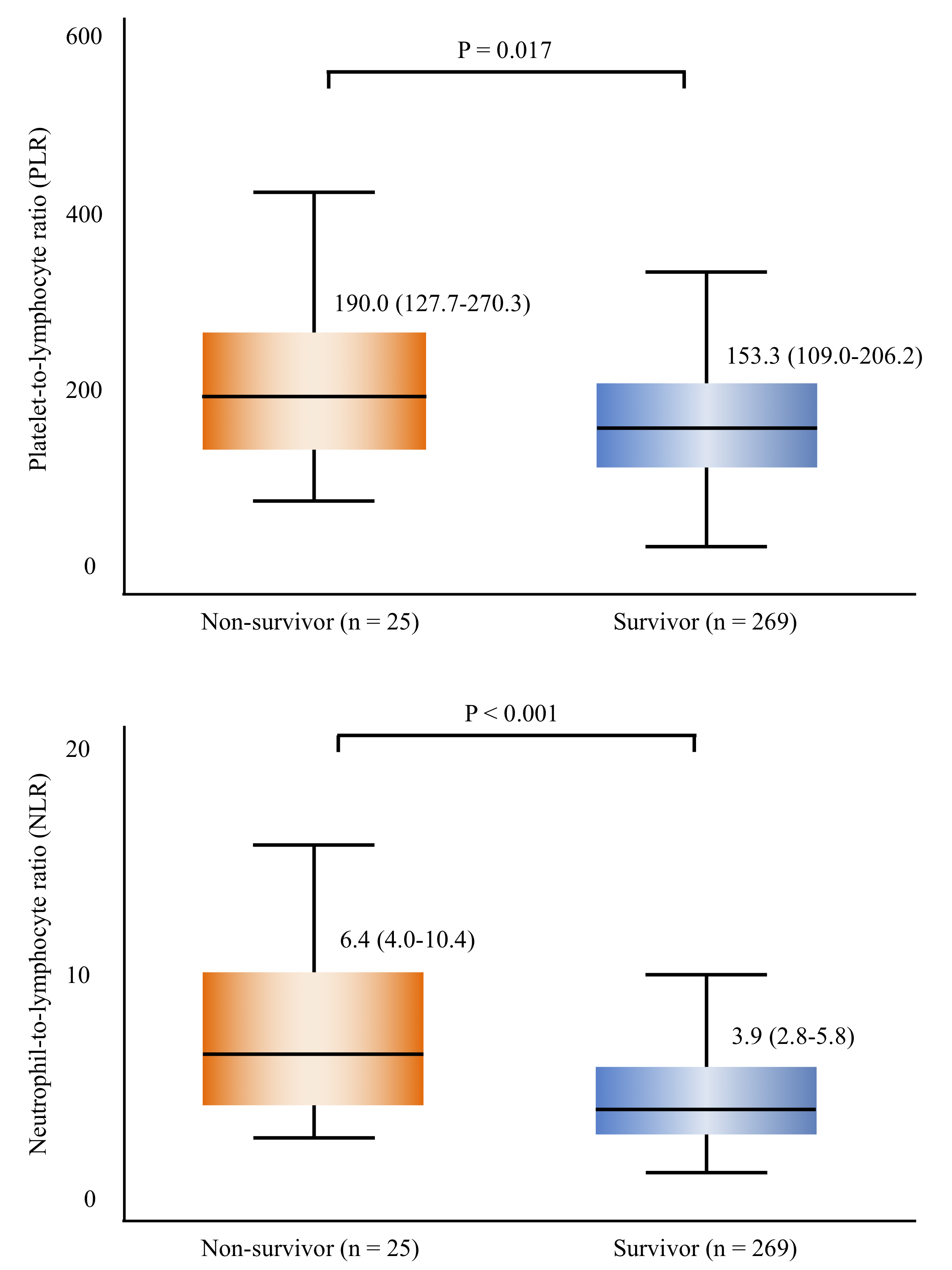

Supplement: Supplementary file 3 — Electronic supplementary material 3 (TIFF 26370 kb) [file 392_2020_1770_MOESM3_ESM.tiff]
