# Supplementary material for: Impact of cancer history on clinical outcome in patients undergoing transcatheter edge-to-edge mitral repair
Source: Clin Res Cardiol. 2020 Nov 9;110(3):440–50. doi: 10.1007/s00392-020-01770-2 (PMC7907025; doi:10.1007/s00392-020-01770-2)
Supplement: Supplementary file 4 — Electronic supplementary material 4 (DOCX 30 kb) [file 392_2020_1770_MOESM4_ESM.docx]

| Supplemental Table 1. Cancer types and past treatment history. | |
| --- | --- |
|  | Total (n = 82) |
| Cancer type (%) |  |
| Breast | 17 (20.7) |
| Colorectal | 17 (20.7) |
| Prostate | 14 (17.1) |
| Leukemia | 14 (17.1) |
| More than one cancer | 5 (6.1) |
| Skin | 3 (3.7) |
| Urinary tract | 3 (3.7) |
| Urinary bladder | 2 (2.4) |
| Kidney | 2 (2.4) |
| Gastric | 1 (1.2) |
| Uterine | 1 (1.2) |
| Thyroid | 1 (1.2) |
| Ovarian | 1 (1.2) |
| Neuroendocrine | 1 (1.2) |
| Lung | 0 (0.0) |
| Head and neck | 0 (0.0) |
| Laryngeal | 0 (0.0) |
| Esophageal | 0 (0.0) |
| Pancreatic | 0 (0.0) |
| Cholangio | 0 (0.0) |
| Gallbladder | 0 (0.0) |
| Adrenal | 0 (0.0) |
| Liver | 0 (0.0) |
| Cancer treatment before TMVR (%) |  |
| Surgery | 50 (61.0) |
| Chemotherapy | 27 (33.0) |
| Radiation therapy | 21 (25.6) |
| unknown | 15 (18.3) |

Abbreviations: TMVR, transcatheter mitral valve repair
