# Supplementary material for: Impact of cancer history on clinical outcome in patients undergoing transcatheter edge-to-edge mitral repair
Source: Clin Res Cardiol. 2020 Nov 9;110(3):440–50. doi: 10.1007/s00392-020-01770-2 (PMC7907025; doi:10.1007/s00392-020-01770-2)
Supplement: Supplementary file 5 — Electronic supplementary material 5 (DOCX 28 kb) [file 392_2020_1770_MOESM5_ESM.docx]

| Supplemental Table 2. The numbers (Kaplan-Meier estimated probabilities) of cardiac death, non-cardiac death and unknown cause of death between cancer and non-cancer group. | | | | |
| --- | --- | --- | --- | --- |
|  | Total  (n = 393) | Cancer  (n = 68) | Non-cancer  (n = 303) | Log-rank P |
| Cardiac death | 12 (3.5) | 4 (5.9) | 8 (2.9) | 0.16 |
| Non-cardiac death | 20 (5.7) | 6 (9.2) | 14 (5.0) | 0.14 |
| Unknown cause of death | 7 (2.4) | 3 (6.6) | 4 (1.5) | 0.075 |
